# A Comparative Study of the Pharmaceutical Properties between Amorphous Drugs Loaded-Mesoporous Silica and Pure Amorphous Drugs Prepared by Solvent Evaporation

**DOI:** 10.3390/ph15060730

**Published:** 2022-06-09

**Authors:** Arif Budiman, Diah Lia Aulifa

**Affiliations:** 1 Department of Pharmaceutics and Pharmaceutical Technology, Faculty of Pharmacy, Universitas Padjadjaran, Jl. Raya Bandung-Sumedang Km. 21, Bandung 45363, Indonesia; 2 Department of Pharmaceutical Analysis and Medicinal Chemistry, Faculty of Pharmacy, Universitas Padjadjaran, Jl. Raya Bandung-Sumedang Km. 21, Bandung 45363, Indonesia; diah.lia@unpad.ac.id

**Keywords:** amorphous, pharmaceutical properties, ritonavir, mesoporous silica

## Abstract

The formulation of poorly water-soluble drugs is one of the main challenges in the pharmaceutical industry, especially in the development of oral dosage forms. Meanwhile, there is an increase in the number of poorly soluble drugs that have been discovered as new chemical entities. It was also reported that the physical transformation of a drug from a crystalline form into an amorphous state could be used to increase its solubility. Therefore, this study aims to evaluate the pharmaceutical properties of amorphous drug loaded-mesoporous silica (MPS) and pure amorphous drugs. Ritonavir (RTV) was used as a model of a poorly water-soluble drug due to its low recrystallization tendency. RTV loaded-MPS (RTV/MPS) and RTV amorphous were prepared using the solvent evaporation method. Based on observation, a halo pattern in the powder X-ray diffraction pattern and a single glass transition (*T_g_*) in the modulated differential scanning calorimetry (MDSC) curve was discovered in RTV amorphous, indicating its amorphization. The *T_g_* was not detected in RTV/MPS, which showed that the loading RTV was completed. The solid-state NMR and FT-IR spectroscopy also showed the interaction between RTV and the surface of MPS in the mesopores. The high supersaturation of RTV was not achieved for both RTV/MPS and the amorphous state due to its strong interaction with the surface of MPS and was not properly dispersed in the medium, respectively. In the dissolution test, the molecular dispersion of RTV within MPS caused rapid dissolution at the beginning, while the amorphous showed a low rate due to its agglomeration. The stability examination showed that the loading process significantly improved the physical and chemical stability of RTV amorphous. These results indicated that the pharmaceutical properties of amorphous drugs could be improved by loaded-MPS.

## 1. Introduction

The aqueous solubility of drugs is important for oral dosage form due to its significant influence on bioavailability [1,2]. Based on the Biopharmaceutics Classification System, over 70% of new drug candidates are poorly soluble in water, causing their low bioavailability and a challenge in the development of new dosage forms [3]. Therefore, developing a strategy to improve solubility is necessary for the formulation of poorly water-soluble drugs, especially in oral dosage form [4].

The amorphous system is a promising strategy in that formulations can improve the dissolution rate and the bioavailability of poorly water-soluble drugs [5]. Several studies have been carried out on the development of amorphous drug formulation in academia and the pharmaceutical industry to overcome poor aqueous solubility [6]. These drugs have a higher Gibbs free energy compared to their crystalline counterparts leading to significantly high solubility and rapid dissolution [7]. The major challenges in the amorphous formulation of API are attributed to the control of its formation and physical stability, which is influenced by the choice of components and the preparation method used [8].

Some APIs are good glass-formers, which are intrinsically easy to be amorphized by cooling or precipitating from a solution. Based on the classification of recrystallization tendency proposed by Taylor et al., amorphous drugs with good glass former did not crystallize upon cooling and reheating [9,10]. Moreover, some drugs have also been marketed in a pure state without any additive compound. Although those with good glass former were stable after cooling and reheating, their thermodynamical instability led to relaxation, nucleation, and crystal growth, especially after dispersion in water and during humidified storage [11,12]. Therefore, the pure drugs are not usually formulated in amorphous preparation but are combined with excipients to stabilize the drug, as well as prevent nucleation and crystallization during the storage or dissolution process [13].

Drug encapsulation into mesoporous silica (MPS) is a promising strategy in the pharmaceutical field due to its ability to stabilize the amorphous form [14]. It can also improve the in vitro dissolution rate and apparent solubility compared to their crystalline counterparts and in vivo performance [15,16]. The two mechanisms that have been proposed for the inhibition of drug crystallization in MS include (1) the adsorption of drugs on MS due to the molecular interaction between the surface of MPS and the functional groups of the drug molecules and (2) the nanoconfinement effect of MPS from a smaller pore diameter compare to critical crystalline nuclei, which caused the suppression of crystal growth [17,18]. This showed that the surface area and pore volume of MS could affect the drug encapsulation and crystallization [19,20].

Several novel types of mesoporous silica materials, such as MCM-41, MCM 48, and SBA-15, have been developed as drug carriers for controlled delivery [21]. SBA-15 is an essential mesoporous substance with a highly ordered hexagonal that has been extensively reported as a drug carrier. The Silanol group’s presence on the surface of SBA-15 can interact with the drug through a weak intermolecular hydrogen bond which is essential for controlling the delivery of drugs [22]. MCM is also an ordered arrangement of cylindrical mesopores. MCM-41 was formed by tubes of silicon ordered in a hexagonal arrangement, while MCM-48 was formed with a cubic arrangement [23].

Although several studies reported the formulation of poorly water-soluble drugs, the comparison of pharmaceutical properties between amorphous drugs within MPS and pure ones has not been clearly understood, particularly for class III drugs. Therefore, it is important to evaluate the comparison of these formulations to determine whether the additive compounds are needed to improve the pharmaceutical properties of the drugs.

This study systematically characterized the amorphous drug with good glass formers within MPS and pure amorphous drugs and also evaluated their pharmaceutical properties. Ritonavir (RTV) was selected as a model of poorly water-soluble drugs in class III. The solvent evaporation method was used to prepare RTV amorphous loaded-MPS and pure RTV, while their molecular state was characterized by powder X-ray diffraction (PXRD), modulated differential scanning calorimetry (MDSC), and solid-state NMR measurements. Furthermore, the solubility test, dissolution profile, physical, and chemical stability were also evaluated.

## 2. Results

**Figure 1:**
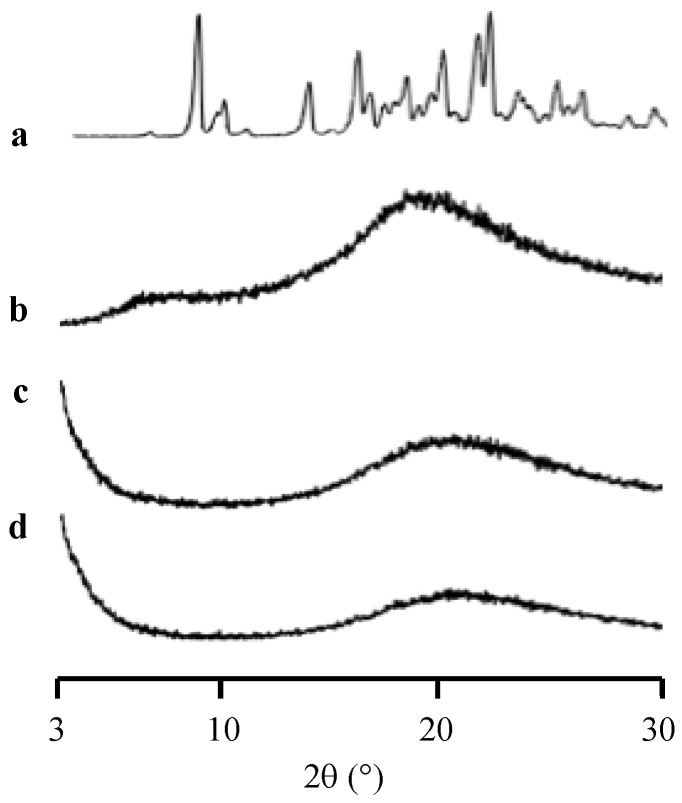
The PXRD patterns of (**a**) RTV crystal, (**b**) RTV amorphous, RTV/MPS = (**c**) 8:2, and (**d**) 3:7.

The PXRD was carried out to characterize the amorphization of RTV after its preparation by a solvent evaporation method (Figure 1). The RTV showed characteristic diffraction peaks in the PXRD patterns which were similar to RTV crystal form II [24,25]. Therefore, the starting material of RTV for this study was a Form II polymorph which is the most stable and least soluble polymorphic form [26]. The RTV/MPS and RTV amorphous showed halo patterns without any characteristic peaks of the RTV crystal. This indicated that the amorphization of RTV was successfully formed by the solvent evaporation method. The PXRD does not detect the presence of an amorphous form; however, it was discovered that the absence of crystallinity in the samples could not be used to confirm the amorphization of the drug by spotting the halo pattern in the diffractogram [27].

**Figure 2:**
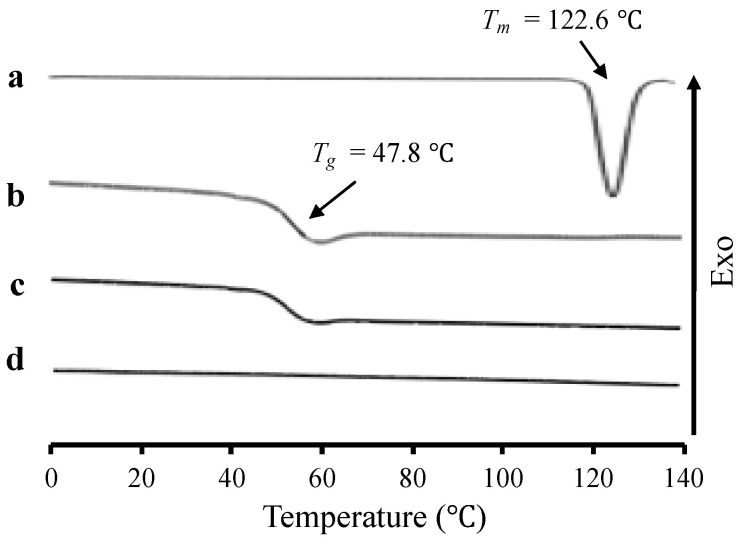
The MDSC curve of (**a**) RTV crystal, (**b**) RTV amorphous, RTV/MPS = (**c**) 8:2, and (**d**) 3:7.

The RTV/MPS and RTV amorphous prepared by the solvent evaporation method were analyzed by MDSC and compared to RTV crystal and MPS material (Figure 2). The RTV crystal showed a melting onset at 114 °C with a peak endothermic of 122.6 °C in the MDSC curve, while its amorphous indicated a glass transition event at 47.8 °C and not a melting peak. These data were in agreement with the previous studies, in which a melting onset at 113 °C with a peak endothermic of 122 °C from RTV was reported [28]. Meanwhile, Baird et al. showed that the glass transition event of RTV was 49 °C [9]. The *T_g_* of RTV was also observed in RTV/MPS = 8:2, indicating that some RTV existed as an amorphous state outside the MPS. Furthermore, there was no RTV melting peak detected for RTV/MPS = 8:2 after heating. This showed that the prepared RTV amorphous was stable, even after heating which is usually observed in class III drugs, based on Taylor’s classification. However, the RTV/MPS = 3:7 showed no glass transition or melting peak after heating, which was indicated by the molecular dispersion of RTV within MPS [29]. The previous studies reported that the absence of a glass transition event could be attributed to the monomolecular adsorption of drugs on the surface of MPS [5,29,30].

**Figure 3:**
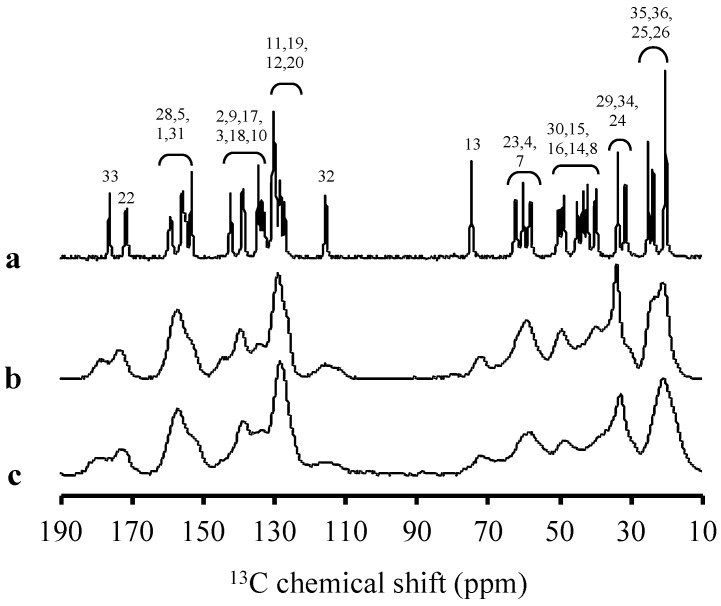
^13^C NMR spectra (υ = 5 kHz) of (**a**) RTV crystal, (**b**) RTV amorphous, and (**c**) RTV/MPS = 3:7.

The solid-state ^13^C CP/MAS NMR was carried out, as shown in Figure 3, to confirm amorphization and evaluate the molecular state of RTV amorphous, specifically within MPS. The peaks of RTV were also assigned through comparison with the solution-state ^13^C NMR spectrum, as reported in a previous study [31]. The peaks of RTV were broadened compared to the RTV crystal, reflecting the wider distribution of the chemical shifts due to the amorphization. The ^13^C CP/MAS spectra of RTV within MPS were in a glassy state, as indicated by their similarity to RTV amorphous. The C5 peak of RTV exhibited a downfield shift from RTV amorphous to RTV/MPS. The difference in the peak shape of the phenyl group (C-11–12, C19–20) at 128 ppm was also observed. These can be attributed to the interaction between RTV and the surface of MPS. However, the relative peak intensities of RTV amorphous at 30 ppm derived from the methyl group (C24–C-26, C-29, C-34–36) were higher than RTV/MPS. This occurs due to the interaction between the RTV and the silica surface of MPS, leading to the change in the local mobility of RTV [32,33].

**Figure 4:**
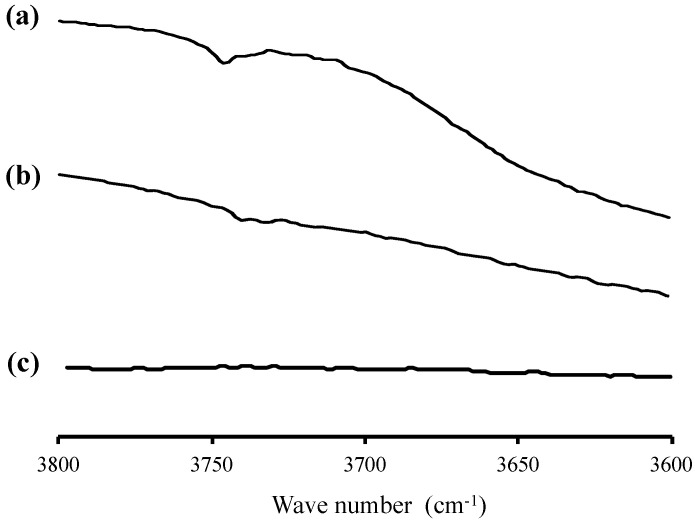
FT−IR spectrum of (**a**) RTV crystal, (**b**) RTV amorphous, and (**c**) RTV/MPS = 3:7, in the OH stretch region.

The interaction between RTV and the silica surface of MPS was further complemented by FT-IR measurement (Figure 4). The characteristic signal around 3750 cm^−1^ which was attributed to the stretching vibrations of isolated, namely non-hydrogen bonded silanol groups, was observed in MPS. Moreover, the bathochromic shift of the silanol group was observed in RTV/MPS, while this spectrum was not detected in the RTV amorphous [34]. This indicated that there is a molecular interaction between RTV and the silanol group on the surface of MPS.

**Figure 5:**
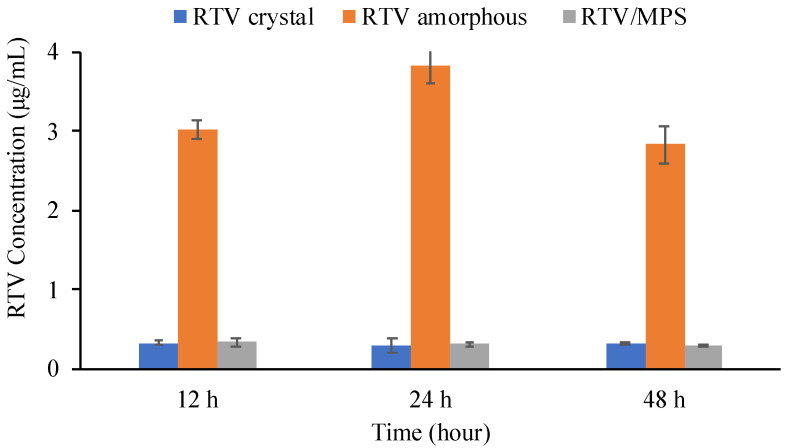
The solubility of each sample at three different times (*n* = 3, mean ± S.D).

**Figure 6:**
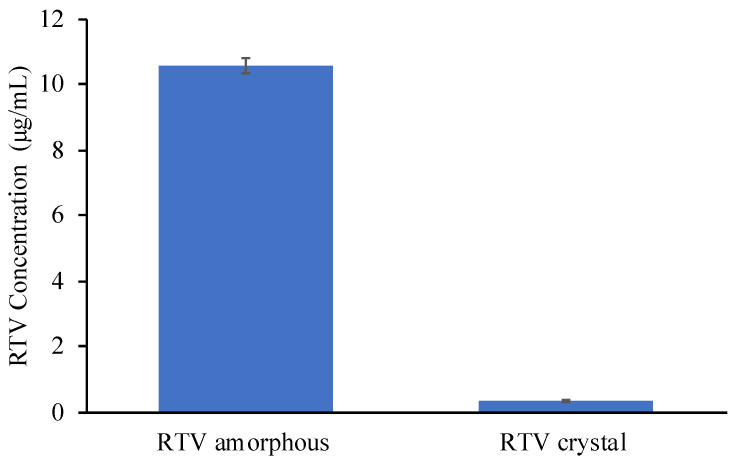
The saturation solubility of RTV amorphous is determined by the centrifugation method (*n* = 3, mean ± S.D).

As shown in Figure 5, the equilibrium solubility of RTV in 50 mM phosphate buffer (pH 6.8, 37 °C) was 0.31 ± 0.03 μg/mL, which is lower compared to the previous study. Meanwhile, the amorphous solubility of RTV measured by centrifugation was 10.58 ± 0.28 μg/mL (Figure 6). The difference in the results is due to the variation in the crystallinity of RTV, the ionic strength in phosphate buffer, and the crystalline polymorph of forms I and II, respectively. The solubility of RTV amorphous measured by the shake flask method showed statistically significant enhancement in the equilibrium solubilities compared to the RTV crystalline. However, the concentration of RTV amorphous was significantly different compared to the solubility measured by centrifugation. The variation in RTV concentration due to recrystallization during measurement by the shake flask method was also observed after 12, 24, and 48 h. Water can easily induce the recrystallization of an amorphous drug. The result of RTV/MPS is almost similar to its crystalline counterparts, even after measurement for 12 h. The RTV was monomolecularly dispersed within MPS leading to its rapid release after dispersion into 50 mM phosphate buffer (pH 6.8, 37 °C). However, the concentration of RTV significantly decreased due to RTV recrystallization in the medium, which indicated that the amorphous almost completely recrystallized after measurement for 12 h.

**Figure 7:**
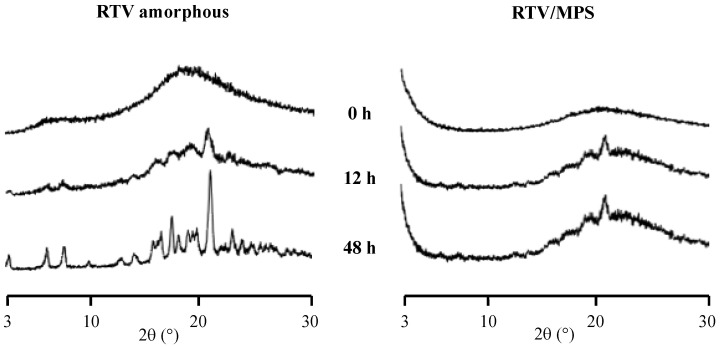
The PXRD pattern of the precipitate following the solubility of each sample in 50 mM phosphate buffer pH 6.8.

The medium of the solubility test was filtered, and the precipitate of the samples was collected and dried using a vacuum for 24 h to confirm the recrystallization of RTV during measurement. Subsequently, the precipitate was evaluated using PXRD, as shown in Figure 7. The characteristic diffraction peaks of RTV crystal were observed in the amorphous state after 12 and 48 h of measurement, indicating the recrystallization of RTV in the medium. A similar result was also observed in RTV/MPS; however, only small characteristic diffraction peaks of RTV crystal were detected. This is due to the lower concentration of RTV compared to MPS. The characteristic diffraction peaks of the RTV crystal were not completely detected. These results showed that the crystallization of RTV appeared during solubility measurement in the medium.

**Figure 8:**
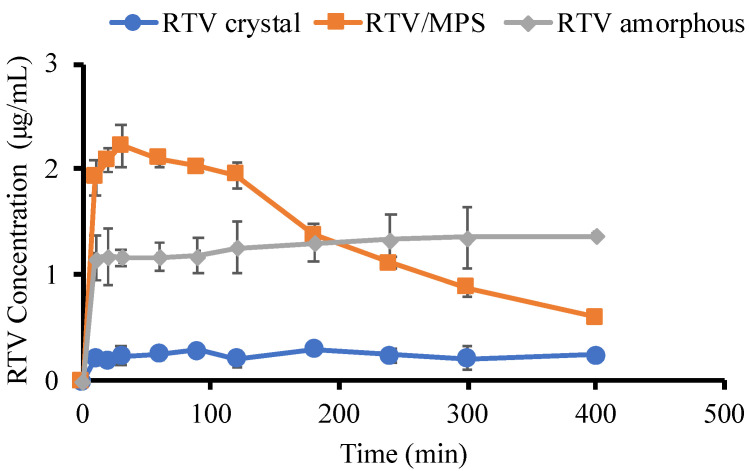
Dissolution profiles of RTV crystal, RTV amorphous, and RTV/MPS = 3:7 in 50 mM phosphate buffer pH 6.8 at 37 °C. (*n* = 3, mean ± S.D).

The dissolution test of each sample was carried out in 50 mM phosphate buffer pH 6.8 at 37 °C under non-sink conditions (Figure 8). The loading efficiency of RTV/MPS = 3:7 was determined as 98.66 ± 1.8% (*w/w*, *n* = 3), where the high loading efficiency was due to the strong interaction between RTV molecules and the functional group of MPS on the silica surface of MPS. In this study, the 50 mM in the dissolution medium was used because a previous study reported that increasing ionic strength would promote the aggregation of molecules through hydrophobic interactions and decrease the coexistence concentration [35]. Furthermore, the RTV crystal showed a slow dissolution rate up to an RTV concentration of 0.24 μg/mL after 400 min. Since the concentration of RTV in the solubility test was 0.31 ± 0.03, the crystalline solubility will be higher than 0.24 μg/mL. This is because the period of the dissolution test (400 min) was not enough to achieve the equilibrium state of the RTV crystal, which has very limited solubility in the phosphate buffer pH 6.8. The dissolution of the amorphous was higher than that of the RTV crystal, and its concentration was around 1.3 μg/mL after 400 min. However, RTV amorphous did not show the “spring-parachute” phenomenon typically observed for amorphous systems, with rapid dissolution at the beginning, followed by a decrease in drug concentration due to recrystallization. The amorphous prepared by solvent evaporation was not well dispersed in the dissolution medium. This caused the formation of large agglomerations upon immediate contact with the dissolution medium leading to a slower rate in line with previous reports [35]. Meanwhile, the dissolution profile of RTV/MPS showed a spring-parachute phenomenon. The rapid dissolution was observed at the beginning of the test and reached a maximum concentration of 2.22 μg/mL after 30 min, which decreased due to associated nucleation and crystallization. Subsequently, their monomolecular dispersion within MPS led to a good dispersibility in the medium at the beginning of the dissolution test. Despite a supersaturated solution, the release of RTV from MPS was incomplete. The amorphous solubility was 10.58 ± 0.28 μg/mL; therefore, the maximum concentration reached by RTV/MPS will be higher than 2.22 μg/mL. A previous study stated that the strong interaction between RTV and the surface leads to an incomplete release of RTV. This indicated that the percentage release of RTV decreases significantly compared to its amorphous solubility.

**Figure 9:**
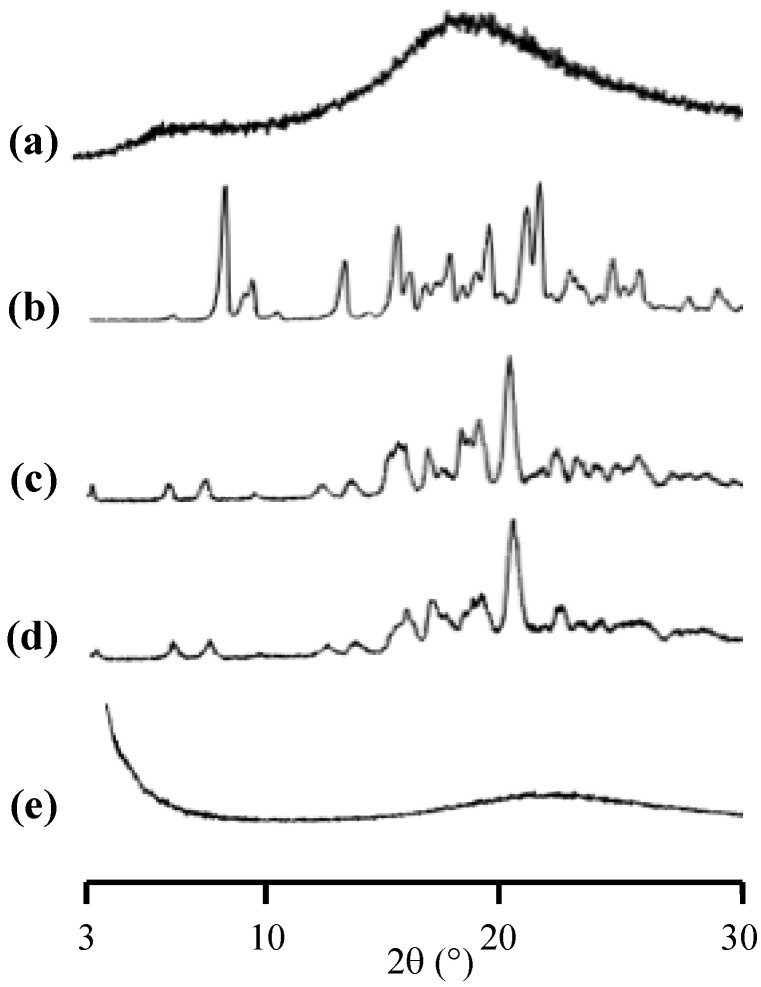
The PXRD patterns of (**a**) RTV amorphous, (**b**) RTV crystal (I), (**c**) RTV amorphous after 7 day storage at 40 °C, 96% RH, (**d**) RTV crystal (II), and (**e**) RTV/MPS = 3:7 after 7 day storage at 40 °C, 96% RH.

The PXRD was carried out to evaluate the physical stability of RTV amorphous and RTV/MPS after storage at 40 °C, 96% RH, as shown in Figure 9. The characteristic diffraction peaks of RTV crystal were observed in its amorphous after 7 days of storage, while RTV/MPS maintained the halo patterns, indicating the retained amorphous state. Despite the presence of humidity and the possibility of partial RTV in the water adsorbed on the solid surface, crystallization was inhibited due to the small pore diameter. This is in line with observation in a previous study, where the recrystallization of drugs encapsulated into MPS will occur when the pore size of MPS is more than 20 times the size of drug molecules [36,37]. Moreover, the interaction between RTV and the surface of MPS was observed, leading to the recrystallization inhibition of RTV. The encapsulation significantly improved the physical stability of RTV amorphous after storage with the presence of humidity.

**Figure 10:**
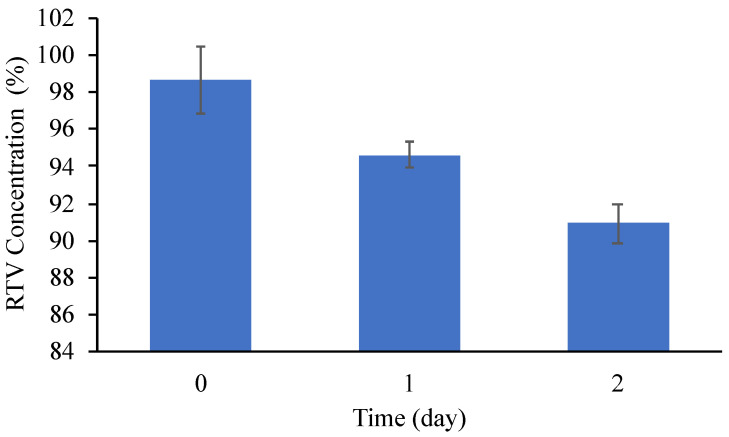
The concentration of RTV from RTV/MPS = 3:7 after 2 day storage at 80 °C, 0% RH (*n* = 3, mean ± S.D).

The effect of temperature on the physical and chemical stability was also evaluated. The samples used were stored in a desiccator containing silica gel at 80 °C, 0% RH. Since the color of RTV amorphous changed from white to yellow, indicating its decomposition, the PXRD measurement and chemical stability test were not carried out. However, the color of RTV/MPS was not changed, and the halo patterns were maintained in PXRD patterns after 2 days of storage at 80 °C (data not shown). The chemical stability was evaluated by measuring the concentration of RTV after storage at 80 °C to confirm the decomposition of RTV within MPS (Figure 10). The result showed that the concentration of the encapsulated drug decreased by around 8% after 2 days of storage due to its decomposition. However, the decomposition rate of RTV/MPS was significantly lower compared to the amorphous state. These results showed that the incorporation of RTV into MPS significantly improved the physical stability and the chemical stability of RTV amorphous.

## 3. Discussion

**Figure 11:**
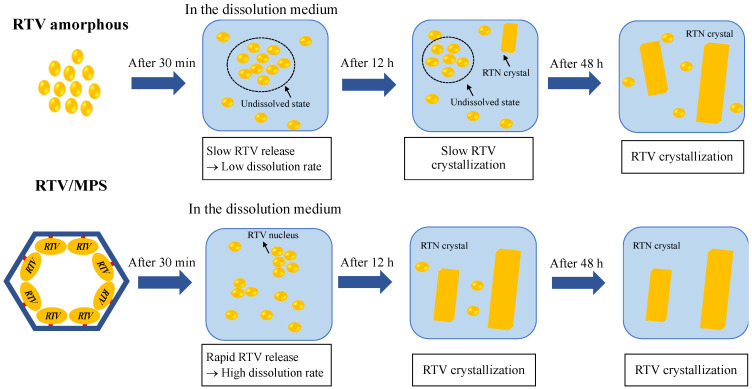
Schematic illustration of RTV amorphous and RTV/MPS in the 50 mM phosphate buffer pH 6.8.

**Figure 12:**
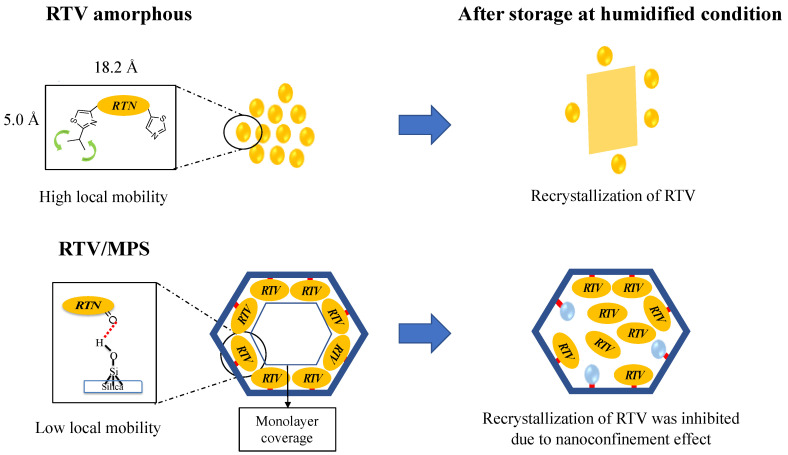
Schematic illustration of RTV amorphous and RTV/MPS after storage in humidified conditions.

A speculated mechanism of the pharmaceutical properties from RTV/MPS and RTV amorphous was proposed in this study, as shown in Figure 11 and Figure 12. The theoretical monolayer coverage of RTV within MPS was determined in the previous report [5]. Based on the theoretical calculation, the capacity of RTV required for the monolayer coverage of MPS was 34.5% (*w/w*). This value is higher than RTV/MPS = 3:7; therefore, it is assumed that all RTV was monomolecularly adsorbed on the surface of MPS, as shown in the MDSC curve. These data were in line with the MDSC curve, where the absence of *T_g_* occurred due to the monomolecular adsorption of RTV on the MPS surface [5,29,30]. The solid-state NMR spectroscopy showed that the local mobility of RTV amorphous within MPS was lower than its amorphous. This is due to the strong interaction between RTV and the surface of MPS. The carbonyl group and phenyl rings of ritonavir also interact with the surface of MPS, as observed in the solid-state NMR experiment (Figure 3). The previous study also stated that the interaction of the drug with the MPS surface restricts their mobility and forms a monolayer [38,39,40]. The hydrogen bonding between the carbonyl group of RTV and the silanol group of MPS was also reported [34]. Therefore, the interactions between RTV and the surface of MPS can decrease the local mobility of the amorphous.

This section discussed the investigation of the mechanism of dissolution from RTV/MPS and RTV amorphous in the 50 mM phosphate buffer of pH 6.8. After dispersion into the dissolution medium, the RTV was rapidly released from MPS and dissolved, leading to a high dissolution rate. Nanosized RTV within mesoporous silica has a larger surface area that improves the wettability of RTV, leading to its high dissolution rate. According to the Noyes–Whitney/Nerst–Brunner equation, the dissolution rate of the drug is increased because the surface area, which is definitely wetted by the solvent, is greater [41]. However, the concentration of RTV gradually decreased because the nucleus and the crystal started forming in the dissolution medium. The high supersaturation of RTV was not achieved after dispersion into the bulk medium. The amorphous solubility of RTV was 10.58 ± 0.28 μg/mL, while the maximum concentration was only 2.22 μg/mL. This occurred due to the strong interaction between RTV and the MPS surface, leading to the incomplete release of RTV from MPS. Although supersaturated solution from drug within MPS was generated and higher than the crystalline state, the percentage release of drug was approximately 20–55% [42]. A previous study also reported that the incomplete release of RTV was hypothesized with a dynamic adsorption equilibrium between RTV adsorbed on the silica surface and free RTV in the solution [34]. Meanwhile, in the case of RTV amorphous, the water only interacts with the surface of the amorphous to form agglomeration after dispersion into the medium. This showed that the amorphous gradually dissolved in the bulk medium, which led to a low dissolution rate. The formation of agglomeration from RTV decreased its surface area leading to the low wettability of RTV; thus, the dissolution rate of RTV amorphous was very low. The high supersaturation of RTV was also not achieved, and the concentration remained unchanged even after being dispersed into the medium for 12 and 24 h, respectively. This occurred because, after 12 h, some RTVs were recrystallized in the bulk medium. Since the RTV amorphous also dissolved gradually, the concentration did not significantly change as the dissolution medium.

The RTV amorphous maintained halo patterns after 30 days of storage at 40 °C, 0% RH (Figure S1). However, at 40 °C and 96% RH, recrystallization was observed after 7 days of storage, which showed that the presence of water content induced the recrystallization of RTV amorphous. The crystal form was also changed from the form II to I after storage in humidified conditions. The difference in characteristic diffraction peaks was detected at 9.5°, 9.8°, 16.1°, and 22.2° 2-theta. A previous study stated that RTV form I was obtained by recrystallization from the solvent [43]. This is because its preparation was almost similar to that of the RTV amorphous. Meanwhile, its recrystallization was not detected in RTV/MPS after storage at 40 °C, 96% RH. The space of MPS was not sufficient to form the RTV nucleus due to a size-constraints effect, which inhibited the recrystallization of RTV. Previous reports stated that amorphous drugs would recrystallize within MPS when the pore size of MPS is 20 times larger than the size of the drug molecule [36,37]. In this study, the molecule size of RTV was 1.82 nm × 1.52 nm [42], while the pore size was 4–5 times larger with an MPS of 8 nm. This showed that the critical nucleus size and further recrystallization of RTV could be efficiently suppressed by the nanoconfinement effect of MPS. Moreover, the hydrogen bonding between the carbonyl group of RTV and the silanol group of MPS could further inhibit the recrystallization of RTV.

## 4. Materials and Methods

### 4.1. Materials

**Figure 13:**
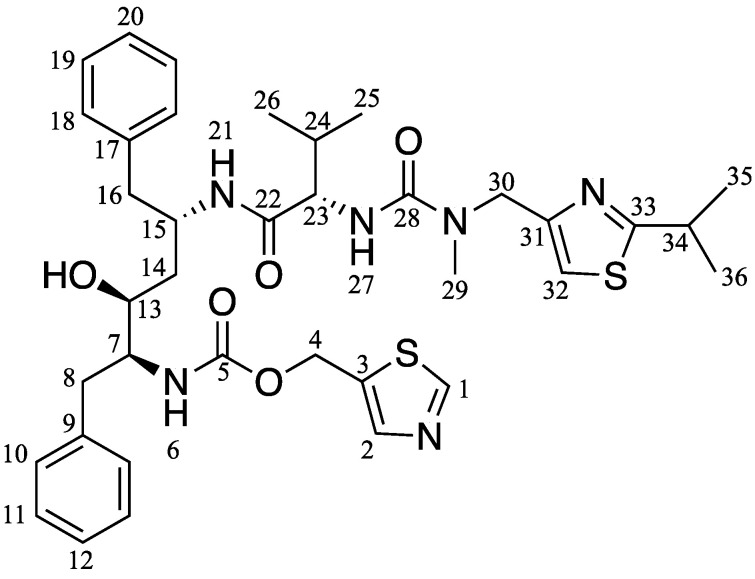
Chemical structures of RTV.

The RTV was purchased from Ontario Chemicals Inc. (Guelph, ON, Canada), and its chemical structures are shown in Figure 13. Furthermore, the MPS was supplied from Taiyo Kagaku., Ltd. (Yokkaichi, Japan). The pore volume, specific surface area, and pore diameter from MPS were 0.92 cm^3^/g, 820 nm^2^/g, and 8 nm, respectively, while its characterization can be seen in Figure S2. Before use, it was ground by mortar and pestle, sieved, and dried at 120 °C for 3 h to remove water content.

### 4.2. Preparation of RTV Encapsulated into MPS

A 60 mg RTV was dissolved in 20 mL chloroform, and 140 mg MPS were dispersed in the solution of RTV. The suspension was sonicated for 3 min using an ultrasonic bath at 25 °C and evaporated for 30 min using a rotary evaporator at 30 °C. The residual solvent was completely removed at 30 °C for 48 h using vacuum drying to obtain RTV encapsulated into MPS (RTV/MPS). A similar method was also conducted for RTV/MPS with a weight ratio of 2:8. The drug loading efficiency was determined using the method described below.

### 4.3. Preparation of the Pure RTV Amorphous

The pure RTV amorphous were prepared by a solvent evaporation method using chloroform as a solvent. The RTV crystal was dissolved in chloroform and evaporated for 30 min using a rotary evaporator at 30 °C. The residual solvent was completely removed at 30 °C for 48 h using vacuum drying to obtain the pure RTV amorphous (RTV amorphous).

### 4.4. Powder X-ray Diffraction (PXRD)

PXRD measurements were carried out using Miniflex II (Rigaku Co., Ltd., Tokyo, Japan) based on the following experimental conditions, target Cu, filter Ni, voltage 30 kV, current 15 mA, scanning rate 4°/min, and scanning angle of 2θ, 5–30°.

### 4.5. Modulated Differential Scanning Calorimetry (MDSC)

The MDSC measurement was carried out using a DSC-7000X instrument (Hitachi High-Tech Science Corporation; Tokyo, Japan). Subsequently, the powder sample with a mass of approximately 5 mg was placed into a crimped aluminum DSC pan under an N_2_ purge at a 50 mL/min flow rate. The glass transition temperature (*T_g_*) was determined at a heating rate of 2 °C/min with modulation of ±0.5 °C every 60 s.

### 4.6. Solid-State NMR Spectroscopy

Solid-state NMR measurements were carried out using the ECX-400 NMR system (9.4 T, JEOL Resonance Inc., Tokyo, Japan) equipped with a JEOL 4 mm HXMAS probe. The 13C NMR spectra were acquired by the cross-polarization (CP)/magic-angle spinning (MAS)/total suppression of spinning sidebands (TOSS) techniques. The measurement conditions include contact time of 2 ms, relaxation delay of 2 s, spinning rate of 5 kHz, and scans 55,000 (~24 h). Hexamethylbenzene was used as an external reference by setting the methyl peak at 17.3 ppm.

### 4.7. Fourier Transform-Infrared (FT−IR) Spectroscopy

The FT−IR was measured by the KBr tablet method using the FT−IR spectrometer (Bruker Optik GmbH, Ettlingen, Germany). FT−IR spectra were determined at a resolution of 4 cm^−1^ in the scan range of 400–4000 cm^−1^.

### 4.8. Solubility Test

Crystalline solubility of RTV, RTV/MPS, and RTV amorphous were determined in 50 mM phosphate buffer pH 6.8 by equilibrating an excess of crystalline RTV in an agitating water bath set at 37 °C for 12, 24, and 48 h. The undissolved samples were separated from the solution by filtration through a 0.45-μm membrane filter. The samples were diluted with acetonitrile and analyzed via high-performance liquid chromatography (HPLC) using the method described below. The amorphous solubility of RTV was also determined by centrifugation, as described in the previous report by Dening et al. (2018) [42].

### 4.9. Drug Loading Efficiency

The drug loading efficiency of RTV was determined by dispersing 20 mg of RTV/MPS into 40 mL methanol with stirring for 2 h. The supernatant (5 mL) was withdrawn, filtered with a 0.45-μm membrane, diluted with acetonitrile, and analyzed by HPLC using the method described below.

### 4.10. Dissolution Test

Dissolution measurements of RTV/MPS and RTV amorphous were carried out by the paddle method using an NTR-VS6P system (Toyama Sangyo Co., Ltd., Osaka, Japan). The samples were dispersed in a 50 mL beaker containing 40 mL of 50 mM phosphate buffer pH 6.8 at an RTV concentration of 50 μg/mL. The buffered solution was equilibrated and stirred with a paddle rotating at 150 rpm. Dissolution was monitored for over 400 min, and 1 mL of the media was withdrawn at fixed time points. The samples were filtered with a 0.45-μm membrane filter, diluted with acetonitrile, and analyzed via high-performance liquid chromatography (HPLC).

### 4.11. High-Performance Liquid Chromatography

HPLC analysis was carried out using a Shimadzu LC 10 AD (Shimadzu Co., Ltd., Tokyo, Japan) equipped with an Inertsil ODS C18 (6.0 × 150 mm) column. The mobile phase consisted of acetonitrile and water at a ratio of 7:3, with a flow rate of 0.7 mL/min. The HPLC was analyzed by injecting 30 μL of each solution into a column maintained at 35 °C, and ultraviolet (UV) detection was at 240 nm.

### 4.12. Physical Stability

Physical stability tests were carried out by storing each sample under different conditions, namely (a) 60 °C and 0%, (b) 40 °C, and 75%. The samples were monitored via PXRD after 0, 4, 7, and 30 days.

### 4.13. Chemical Stability

The chemical stability tests were also carried out by storing each sample at 80 °C and 0% RH. Subsequently, the samples were dispersed into 40 mL methanol with stirring for 2 h. The supernatant (5 mL) was withdrawn, filtered with a 0.45-μm membrane filter, diluted with acetonitrile, and analyzed by HPLC.

## 5. Conclusions

The comparison of pharmaceutical properties between RTV amorphous encapsulated into mesoporous silica (RTV/MPS) and pure RTV was evaluated by the solvent evaporation method and confirmed using PXRD and MDSC measurements. The strong interaction with the surface of MPS in RTV/MPS and the formation of agglomeration from RTV amorphous led to the incomplete release of RTV at the beginning of the dissolution test. The nanoconfinement effect of MPS inhibited the recrystallization within MPS and postponed the decomposition of RTV after storage in humidified conditions and high temperatures. This indicated that the encapsulation of RTV amorphous into MPS significantly improved the pharmaceutical properties of RTV, although the amorphous was stable at ambient temperature. This study provided basic information on the formulation of the amorphous drug with good glass formers in class III.

## Data Availability

The original contributions presented in the study are publicly available.

## Supplementary Materials

The following supporting information can be downloaded at: https://www.mdpi.com/article/10.3390/ph15060730/s1, Figure S1: The PXRD patterns of RTV amorphous after (a) 0, (b) 7, (c) 14, (d) 21, and (e) 28 day-storage at 40 °C and 0% RH. Figure S2: The characterization of MPS after being evaluated by (a) DSC, (b) XRD, (c) FT-IR, and (d) TEM.
