# Supplementary material for: A Comparative Study of the Pharmaceutical Properties between Amorphous Drugs Loaded-Mesoporous Silica and Pure Amorphous Drugs Prepared by Solvent Evaporation
Source: Pharmaceuticals (Basel). 2022 Jun 9;15(6):730. doi: 10.3390/ph15060730 (PMC9228546; doi:10.3390/ph15060730)
Supplement: Supplementary file 1 [file pharmaceuticals-15-00730-s001.zip › pharmaceuticals-1735877-supplementary.pdf]

Supplementary Material

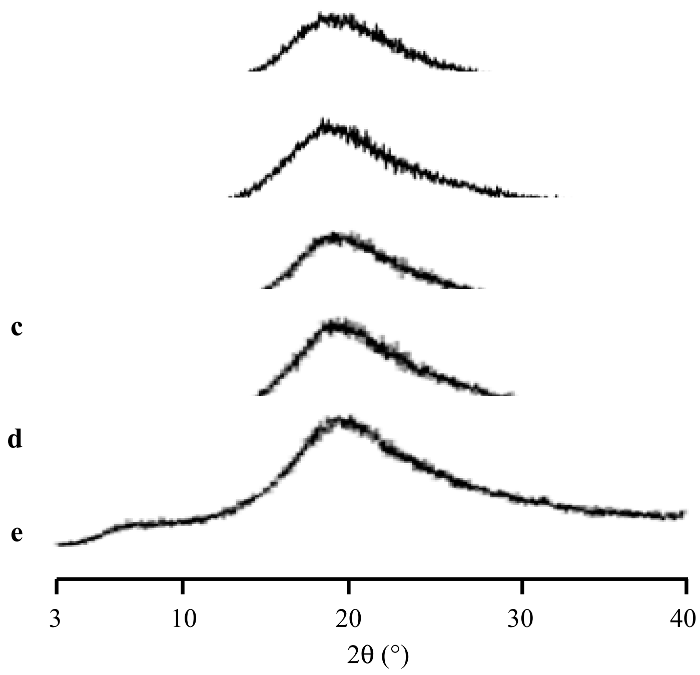

Figure S1. The PXRD patterns of RTV amorphous after (a) 0, (b) 7, (c) 14, (d) 21, and (e) 28 day-storage at 40 ◦C and 0 % RH.

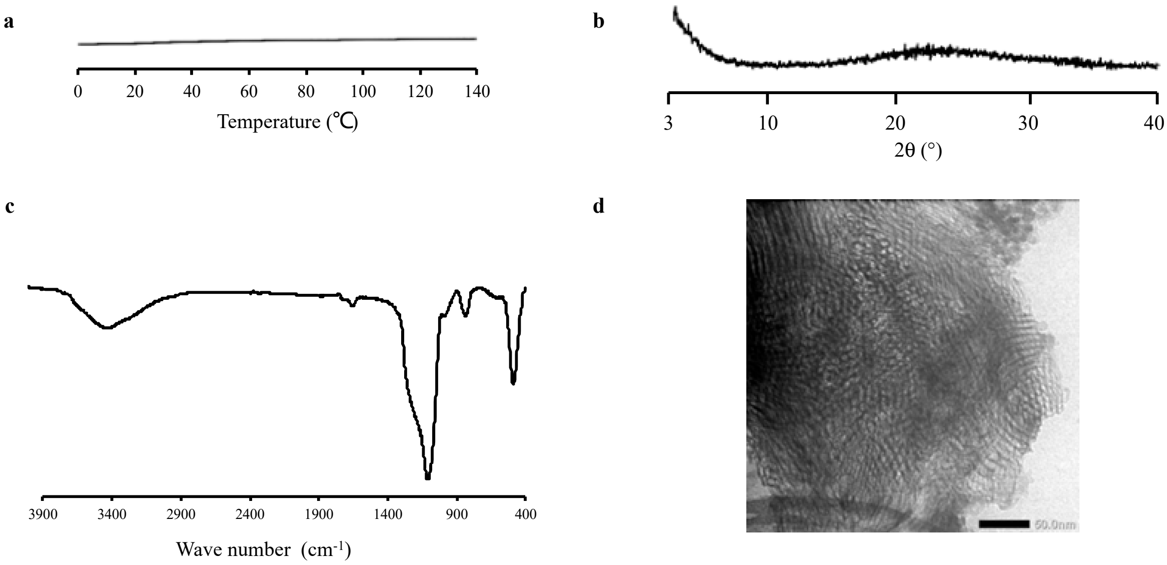

Figure S2. The characterization of MPS after being evaluated by (a) DSC, (b) XRD, (c) FT-IR, and (d) TEM.
